# A dental revolution: The association between occlusion and chewing behaviour

**DOI:** 10.1371/journal.pone.0261404

**Published:** 2021-12-15

**Authors:** Christopher Martin Silvester, Ottmar Kullmer, Simon Hillson

**Affiliations:** 1 Institute of Archaeology, University College London, London, United Kingdom; 2 Department of Paleoanthropology, Senckenberg Research Institute and Natural History Museum Frankfurt, Frankfurt, Germany; 3 Department of Palaeobiology and Environment, Institute of Ecology, Evolution, and Diversity, Johann Wolfgang Goethe University, Frankfurt, Germany; University of Padova: Universita degli Studi di Padova, ITALY

## Abstract

Dentistry is confronted with the functional and aesthetic consequences that result from an increased prevalence of misaligned and discrepant dental occlusal relations in modern industrialised societies. Previous studies have indicated that a reduction in jaw size in response to softer and more heavily processed foods during and following the Industrial Revolution (1,700 CE to present) was an important factor in increased levels of poor dental occlusion. The functional demands placed on the masticatory system play a crucial role in jaw ontogenetic development; however, the way in which chewing behaviours changed in response to the consumption of softer foods during this period remains poorly understood. Here we show that eating more heavily processed food has radically transformed occlusal power stroke kinematics. Results of virtual 3D analysis of the dental macrowear patterns of molars in 104 individuals dating to the Industrial Revolution (1,700–1,900 CE), and 130 of their medieval and early post-medieval antecedents (1,100–1,700 CE) revealed changes in masticatory behaviour that occurred during the early stages of the transition towards eating more heavily processed foods. The industrial-era groups examined chewed with a reduced transverse component of jaw movement. These results show a diminished sequence of occlusal contacts indicating that a dental revolution has taken place in modern times, involving a dramatic shift in the way in which teeth occlude and wear during mastication. Molar macrowear suggests a close connection between progressive changes in chewing since the industrialization of food production and an increase in the prevalence of poor dental occlusion in modern societies.

## Introduction

An increased prevalence of misaligned and crowded dental arches among industrialised groups has been attributed to the consumption of a more heavily extraorally processed diet [1, 2]. Developments in food processing technologies and agriculture have effectively removed the abrasive and fibrous content from the diet of modern humans in industrialised societies [3]. This replaced the coarser dietary pattern of pre-industrial times [4, 5]. A dietary revolution, which swept across Europe during the 18th and 19th centuries, began this process alongside revolutions in industry, agriculture and transportation [6]. By the close of the 19th century, food production had been transformed into a highly mechanised and large-scale industry producing increasingly heavily processed and standardised foods [7–9]. The age of sugar and processed food has continued into the 21^st^ century, characterising the eating habits of vast swathes of the global population, and constituting a marked departure from the foods that were prominent throughout the evolution of hominins [10].

A reduction in jaw dimensions and an increase in poor dental occlusion among modern groups has been discussed as an adaptive consequence of the reduced dental processing of the softer diet that emerged during and following the Industrial Revolution (1760–1840 CE) [11–14]. This is because the upper and lower jaws are highly plastic during development and growth and are shaped by the demands placed upon the masticatory system, principally during chewing [15, 16]. In the absence of sufficient alveolar growth, inadequate space for emerging teeth and disharmonious jaw relationships are anticipated [17–19]. Consequently, it has been hypothesised that underlying these changes are differences in chewing behaviour when compared to pre-industrial groups [1, 2, 14]. This hypothesis, however, remains to be tested.

Clinical feeding studies indicate that human chewing behaviours adapt to the mechanical properties of the food consumed, such as toughness and hardness, as well as their extrinsic surface characteristics, such as stickiness, particle size and roughness [20–23]. Different hardness and toughness properties require different types of jaw movement and tooth-tooth interaction to effect fracture [24, 25]. As foods became increasingly heavily processed in the modern era, changes in chewing behaviours would be anticipated and phenotypic changes in craniofacial structures would be the result. Orthodontic treatment currently focuses on the therapeutic modification of occlusion once often functionally and aesthetically compromised relationships have already developed [26]. Groups consuming coarser and biomechanically demanding food stuffs often exhibit low prevalence rates of malocclusion when compared to industrialised societies; this has been used as evidence to support artificially inducing the environmental conditions these non-industrialised groups experience during development and growth to reduce the likelihood of occlusal problems developing [18, 27]. To address the practicalities of the primary prevention of the development of occlusal problems, differences between the masticatory behaviours of pre-industrial and industrialised human groups need to be investigated.

In humans, the chewing cycle begins with puncture-crushing cycles in which the teeth come together in a vertically directed squashing action comparable to the crushing action of a pestle and mortar [28, 29]. Later in the chewing sequence, as the mass of food is reduced, the surfaces of the teeth come together more closely in a movement called the power stroke. This is divided into two phases. In phase I, the later part of jaw closing, the lower molars on the working side move from a lateral position following an upward, anteriorly and medially directed trajectory terminating in maximum intercuspation [30]. The lower teeth then follow an anterior, medially and slightly downward directed movement during phase II of the power stroke which is followed by jaw opening [30, 31].

During the power stroke, a layer of particles suspended in saliva, likely including a combination of food particles, abrasives and detached enamel, is present between the tooth surfaces. This results in the development of dental wear facets, highly polished planar surfaces, at specific areas of the occlusal surface against which abrasive particles are repeatedly moved across and trapped against as the dental surfaces slide past one another during the power stroke. Each wear facet that develops as a result of the masticatory power stroke has a corresponding wear facet in the teeth of the opposing dental arch [32, 33]. The jaw movements responsible for the creation of dental wear facets during the chewing cycle can be reconstructed from their orientation and inclination [30, 34, 35]. Wear facets have been used to infer the inclination and orientation of the occlusal portion of the chewing cycles of extinct and extant mammals [36, 37], and to reconstruct occlusal relationships [34], and to compare the dietary and masticatory behaviours of hominids [38–41]. Wear facets may also result from para-masticatory activities such as bruxism, the repetitive grinding or clenching, or also by the use of teeth as a third hand [42].

Bread was the principal dietary staple in both the pre-industrial and industrial groups. In the medieval period, cereals formed up to 80% of calorific intake [43]. Similarly, the lower classes in the 19th century typically subsisted on a monotonous diet of bread, potatoes, sugar and sweetened tea [7, 44–46]. Despite this, the physical properties of the bread eaten were dramatically altered by the technological developments in milling that occurred during the Industrial Revolution. In the medieval period, grains were pulverised to form a coarse mixture containing all parts of the grain, including a good deal of finely ground bran and most of the germ, and a high quantity of abrasive particles were retained in the final flour [8, 47]. This resulted in loaves that were coarse, hard and tough when compared to the bread consumed during the Industrial Revolution [43]. Developments in grinding and sieving techniques within the milling process meant that by the close of the 19th century soft white wheaten bread made from finely milled flour became accessible to all social classes [44]. For this reason, it would be anticipated that chewing behaviours and dental wear patterns in the industrial period would reflect this shift towards the consumption of softer and more heavily processed dietary staples when compared to their pre-industrial counterparts.

The aim of this paper is to determine whether changes in chewing behaviour occurred as a result of the consumption of more heavily processed staple foods during the Industrial Revolution. A method of three-dimensional dental macrowear pattern analysis [30, 35, 48], Occlusal Fingerprint Analysis (OFA), was used to compare the molar macrowear patterns and to reconstruct the occlusal behaviours of individuals from the industrial era (1,700–1,900 CE) and a pre-industrial group dating to the medieval and early post-medieval periods (1,100–1,700 CE). Static OFA was used to analyse the molar macrowear patterns [48], and the dynamic OFA [30] was performed to reconstruct occlusal power stroke kinematics for testing the following null hypotheses: (1) there are no significant differences in wear facet area proportions, and wear facet inclinations are consistent in lower second molar macrowear patterns between the pre-industrial and industrial group; (2) the power stroke trajectories and the development of occlusal contact areas are similar between the two groups; (3) there are no significant differences in occlusal topography between the groups; (4) potential confounding factors such as age-at-death and sex of an individual do not impact the molar macrowear patterns observed.

An association between changes in chewing behaviour and the increased prevalence of poor occlusion evident in industrialised societies may inform functional and preventative treatments in the context of contemporary dental practice.

## Materials and methods

### Selection of skeletal assemblages

**Fig 1:**
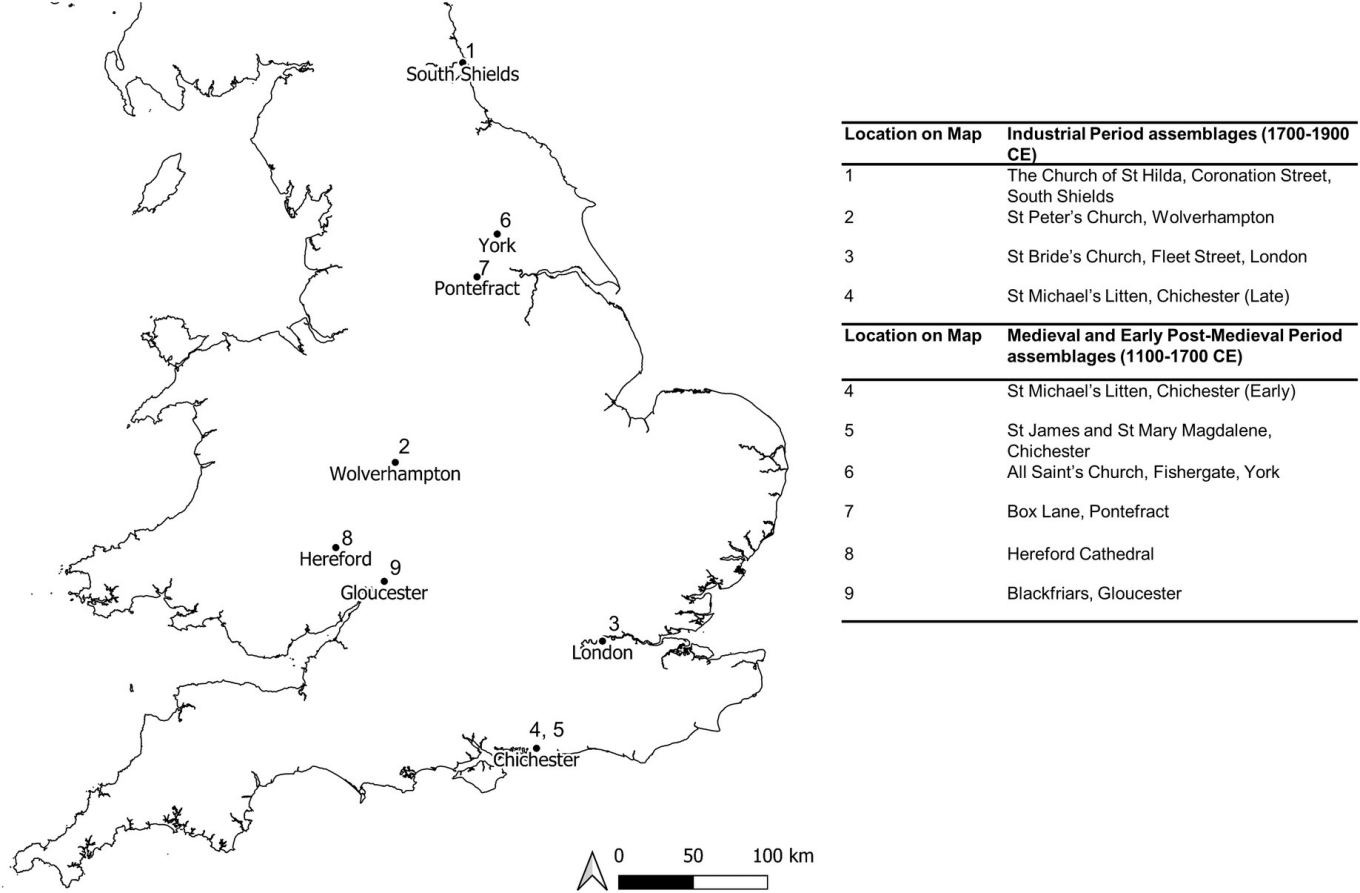
Map showing the location of the cemetery assemblages examined in the current research. Assemblages were excavated at towns and cities across England and represented a variety of burial contexts, including lay cemeteries, those associated with monasteries and a leper hospital. Cemeteries with an approximate date range are indicated by circa (c.). Contains OS data © Crown copyright [and database right] [2021].

Specimens were selected from five British cemetery assemblages dating to the medieval and early post-medieval periods (n = 130) and from four industrial era British cemeteries (n = 103) (Fig 1). The ethical guidelines for handling human remains outlined by UCL, the University of Bradford, University of Sheffield and Museum of London were adhered to during data collection (https://www.ucl.ac.uk/archaeology/research/ethics, https://www.bradford.ac.uk/archaeological-forensic-sciences/facilities/barc/BARC_human_remains_policy.pdf, https://www.sheffield.ac.uk/polopoly_fs/1.573395!/file/Guidelines_for_conduct_in_osteology_labs.pdf, https://www.museumoflondon.org.uk/application/files/5714/8129/0350/Museum_of_London_Policy_for_the_Care_of_Human_Remains.pdf). No permits were required for the described study beyond the written permissions given by the individual institutions responsible for the human remains. The study complied with all relevant regulations.

The principal research question sought to identify differences in masticatory behaviours following the introduction of more heavily processed and softer dietary staples in the industrial period. Historical and archaeological evidence indicated that the majority of assemblages selected were largely representative of the average diet and lifestyle in either the medieval, early post-medieval or industrial periods (Table 1). A range of socioeconomic groups and regions were represented by the assemblages selected in order to encompass a portion of the social and dietary variability described within each period across England. The two study groups were built up as follows:

- Each assemblage was dated to either the medieval and early post-medieval periods (1100–1700 CE) or the industrial period (1700–1900 CE). Where inhumations at a single cemetery overlapped both periods, contextual information for each burial had to be adequate to disentangle the earlier from the later burials. The only cemetery selected that required separation into early and later phases was St Michael’s Litten, Chichester (ESC11), which dated from 1550–1900 CE.
- Sufficient contextual information for each assemblage had to be available to characterise the general profile of the individuals interred at the site.
- Most of the assemblages included were selected because they were drawn from cemeteries of a substantial size (greater than 100 individuals) and likely reflected the average demographic for the period and region.
- If assemblages from approximately the same geographic region were available in the medieval, early post-medieval and industrial periods these assemblages were prioritised.
- The assemblage had to be available for examination within a suitable timeframe for data collection to be completed (September 2017 to September 2019).
- At least five individuals within the assemblage had to satisfy the individual selection criteria below. Larger assemblages were prioritised to increase the likelihood that this condition would be met.

**Table 1:** Table showing the assemblages included in the current study and giving details of their locations, size, date and the number of individuals from this collection that satisfied the inclusion criteria. Individuals from cemeteries dating from 1100–1700 CE formed the pre-industrial group and those from cemeteries dating from 1700–1900 CE formed the industrial group.

Collection	Location of Collection	Dates Cemetery in Use (CE)		Number Suitable/ Number of Individuals in Collection		Type of Group		Publications in which Assemblage is described
**St Michael’s Litten, Chichester (Late)**	UCL	c. 1700–1900		26/300		Urban		[49]
**St Bride’s Church, Fleet Street**	St Bride’s Church	1714–1848		31/227		Urban parish burial ground		[50]
**The Church of St Hilda, Coronation Street, South Shields**	University of Sheffield	1816–1856		25/114		Urban working-class group; ship builders		[51]
**St Peter’s Wolverhampton**	University of Bradford	1800–1853		18/150		Urban; short burial period		[52]
**Industrial Group**	**Total**		103					
**St Michael’s Litten, Chichester (Early)**	UCL	c. 1550–1700		18/300		Urban		[49]
**All Saint’s Church, Fishergate, York**	University of Sheffield	c. 1100–1500		32/547		Lay cemetery		[53]
**Hereford Cathedral**	University of Bradford	c. 1100–1600		42/1200		Lay cemetery including two large plague pits		[54]
**Box Lane**	University of Bradford	c. 1100–1500		7/88		Lay Cemetery		[55]
**Blackfriars**	University of Bradford	1246–1539		9/192		Friary		[56]
**St James and St Mary Magdalene, Chichester, West Sussex**	University of Bradford	c. 1100–1600		24/374		Leprosarium and alms house		[57]
**Pre-Industrial Group**	**Total**		130					

### Background information on the cemeteries

Many of the individuals examined dating to the medieval period were derived from lay cemeteries. The cemetery of All Saint’s Church, Fishergate, was situated south of the medieval city walls of York on the east side of the River Foss. The occupants of the cemetery were likely civilians drawn from a range of socioeconomic groups. It was likely established in the late 11th century and fell out of use shortly after 1585 CE [53]. The skeletons examined from Hereford Cathedral were derived from the excavation of the area west of the Bishop’s Cloister. Most burials were probably interred from the 12th to the 16th centuries. The cemetery area might be associated with the parish of St John. St John’s parish lacked a parish church but included small parcels of land across the city, including the city centre. As a result, the burial area would represent all social classes drawn from across the city [54].

Other medieval cemetery assemblages assessed also included monastic burials. The cemetery at Box Lane, Pontefract was likely associated with St John’s Priory, which was founded in about 1090 CE. Pottery recovered supports burial dates from the 13th to the 14th century. It is uncertain whether the cemetery served the Cluniac monks themselves or the lay population of the monastery and the community of nearby settlements. The cemetery included both women and men, with relatively few children, infants and adolescents, supporting the latter interpretation. Burials were modest and bodies were likely placed in shrouds without the use of coffins [55]. The assemblage from Blackfriars, Gloucester was associated with the Dominican Friary established in 1239 CE. The Blackfriars were known for the relative poverty of their lifestyles [58]. The burials included the friars themselves, including a priest with his pewter chalice and paten, in addition to women and high numbers of infants and children. This suggests that benefactors to the Friary and their families were also interred in the burial ground. Furthermore, it is possible that the friars may have operated a hospital at least until the late 15th century and hospital patients may have been buried at the Friary [56].

A subset of the medieval material analysed was associated with the medieval leprosarium and later post-medieval almshouse of St James and St Mary Magdalene, Chichester, likely dated from the 12^th^ to the 17^th^ centuries. Individuals with leprosy and males were concentrated in the southwestern portion of the excavation area whilst females, subadults and non-leprous individuals became more frequent in the northern area of the site. It has been proposed that the earliest portion of the cemetery is the southwestern portion where the prevalence of leprosy is greatest [57].

The industrial group was similarly drawn from a wide socioeconomic spectrum and included London, Chichester and the more northerly industrial centres of Wolverhampton and South Shields. The Litten cemetery, encountered during the excavation of Eastgate square, Chichester, was estimated to have been in use from 1100–1850 CE. Although, most burials date from the 17th-19th centuries. This cemetery likely represented a broad cross-section of the socioeconomic groups present at Chichester and could be divided between the pre-industrial and industrial groups in the current research using burial type [49]. The earlier burials within this cemetery were likely contemporaneous with those inhumed at the leprosarium of St James and St Mary Magdalene, Chichester [59]. The material from St. Bride’s church London were derived from within the church and they were accompanied by lead coffin plates giving name, age and date of death. The first individual was born in 1696 CE and the last in 1852 CE. A large proportion of the individuals interred at St Bride’s were likely workers involved in trade and crafts along the Thames, but burial records indicate that the status of the individuals interred varied greatly, ranging from the lord mayor to individuals in poverty. Qualification for burial at St Bride’s church may have principally depended on location rather than social status [50]. The assemblage from Coronation Street, South Shields, was associated with the Church of St Hilda and dated from 1816–1860 CE. Most of the individuals were interred in less expensive mass-produced coffins indicating that they were probably working class. Variability in coffin furniture and burial situation suggested the cemetery group occupied a relatively wide range of financial statuses. The richer burials at the site were less ornate, however, than most other contemporary middle- and upper-class crypts [51]. The assemblage derived from the excavation of the 19th century overflow burial ground for St Peter’s Collegiate Church, Wolverhampton, was dated to 1830–1880 CE based on the style of coffin fittings. This corresponded to a period of rapid urban growth in Wolverhampton as it developed as a manufacturing centre. Many people were involved in ironmaking and other workshop activities [52].

The skeletal assemblages examined were drawn from a range of geographic locations and social backgrounds within England. Consequently, post-hoc pairwise testing was performed to determine whether any differences observed between the two groups were particularly driven by any of the assemblages examined. These factors are explored further in [60].

### Selection of specimens and sample demography

Second lower molars were selected for OFA as it has been shown to provide an effective representation of masticatory behaviours in primates [61]. OFA can only be effectively applied to moderately worn teeth because dental wear facet patterns are obliterated at more advanced stages of wear [39]. Unworn teeth are similarly uninformative. Thus, lower second molars had to show wear corresponding to Smith score 3 [62] (moderate cusp removal with or without a maximum of pin-point dentine exposure).

Individuals were prioritised if they had relatively good representation of the dentition, preferably with some representation of both the upper and lower dental arcades. The best-preserved side was chosen and in cases of an equivalent state of preservation the right side was analysed. Dental pathology, including ante-mortem tooth loss and dental caries, was present in many of the dentitions examined. Carious lesions were marked as present if a cavity involving the crown was observed on the tooth. The impact of these confounding factors (sidedness, carious lesions, ante-mortem tooth loss) was previously assessed [60] and considered negligible for the current study.

Individuals for whom sex and age-at-death estimates could be made were also prioritised. Sex was estimated by assessing the morphology of the pubic bone, cranium and greater sciatic notch of the innominate bone [63, 64]. The assemblages in both periods were comprised of a mixture of males and females (Table 2). Age-at-death was estimated entirely from the skeleton, in order to make it independent from the development of the dentition. It was based on an assessment of degenerative changes in the auricular surface of the ilium [65]. This method was selected because the pubic symphysis was commonly absent or poorly preserved. The pre-industrial group was dominated by a larger number of individuals from the younger age-at-death category (Buckberry-Chamberlain score ≤9) (Table 3). Sex and age-at-death were also assessed as factors which may have contributed to the variability in wear facet patterns observed.

**Table 2:** Sex distribution of the individuals selected from the assemblages examined and used to perform OFA.

Site	Indeterminate	Female	Male
**York Barbican**	17	8	7
**Blackfriars, Gloucester**	4	2	3
**Box Lane, Pontefract**	4	3	0
**St James and St Mary Magdalene, Chichester**	3	13	8
**Hereford Cathedral**	16	14	12
**Medieval Summary**	44	40	30
**Coronation Street, South Shields**	7	6	12
**St Michael’s Litten, Chichester**	0	19	27
**St Bride’s, London**	0	13	18
**St Peter’s, Wolverhampton**	5	5	8
**Industrial Summary**	12	43	65

**Table 3:** Age-at-Death distribution using the Buckberry-Chamberlain method (2002) of the individuals selected from the assemblages examined and used to perform OFA.

Age Category	Unknown	Younger	Older
**Buckberry-Chamberlain score**	**-**	**≤9**	**≥10**
**Site**			
**York Barbican**	3	25	4
**Blackfriars, Gloucester**	1	8	0
**Box Lane, Pontefract**	2	5	0
**St James, Chichester**	0	24	0
**Hereford Cathedral**	13	28	1
**Medieval Summary**	19	90	5
**Coronation Street, South Shields**	3	11	11
**St Michael’s Litten, Chichester**	1	35	10
**St Bride’s, London**	0	15	16
**St Peter’s, Wolverhampton**	6	7	5
**Industrial Summary**	10	68	42

To conduct dynamic OFA analysis, one individual was selected from the pre-industrial group and one from the industrial group to simulate power stroke kinematics. Individuals were selected with adequate preservation of the upper and lower tooth rows and who presented lower second molar wear facet patterns characteristic of each period; their wear facet area proportions approximated the centre values for either the pre-industrial or industrial group. 3D models of antagonistic upper and lower molar rows were required and generated using the methods outlined below.

### Generation of occlusal models

A dental gypsum model of one of the lower second molars was produced for each individual. The reflective and lustrous qualities of the enamel rendered the direct generation of 3D models from the original dental surfaces problematic [66], therefore dental impressions were taken. The molar row was first gently cleaned using acetone prior to an impression being taken. An impression of each lower second molar was taken using a two-phase, two-step, putty-wash technique utilising President® putty soft and President® light body polyvinylsiloxane impression materials (Coltène/Whaledent Inc) [67–69]. Dental casts were made using non-reflective dental die stone (Suprastone® dental die stone type IV; Kerr Corporation). Virtual dental models were generated using a structured light scanning system (GOM ATOS 80 scanner, GOM, Braunschweig, Germany). Data acquired was imported directly into ATOS professional (v 2018 Hotfix 3) and then converted into a polygonal surface mesh, which could be exported in stl format for analysis.

### Occlusal fingerprint analysis

**Fig 2:**
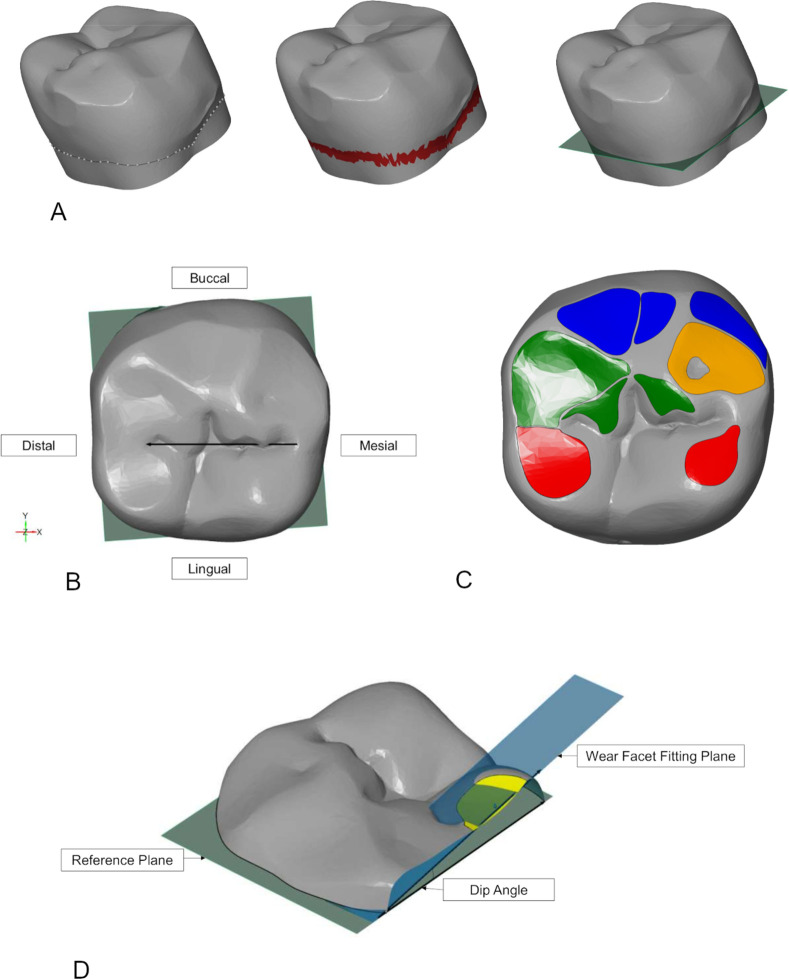
Process of conducting occlusal fingerprint analysis in GOM Inspect. A) Process used to create the reference plane through the cervix of the tooth. B) Correct alignment of a lower left molar using the cervical reference plane and mesio-distal axis of the tooth. C) A tooth with the wear facets demarcated using surface curves and colored according to their functional role during the power stroke: blue indicates buccal phase I, orange corresponds to lingual phase I, green to phase II and brown is a tip crushing area. The orientation of the tooth is the same as in diagram B. D) Illustration of how dip angle was calculated. The angle between a best-fit plane fitted to the wear facet and the cervical reference plane was measured to give the dip angle.

Wear facet analysis was performed using GOM Inspect (Version 2018 Hotfix 6) based on the method of Kullmer *et al*. [48] (Fig 2). The teeth were consistently orientated in the software using a gaussian best-fit plane attached to the cervical margin of the tooth [48, 70]. This was created by drawing a curve around the cervix of the tooth and selecting an area 0.2mm above and below the curve. Wear facets on the occlusal surface of each lower second molar were identified, demarcated and labelled using the terminology of Maier and Schneck [71, 72], modified after Kullmer et al. [48]. The area and inclination of each wear facet was measured and grouped according to the phase of the power stroke sequence with which it was associated, either phase I (divided into buccal phase I and lingual phase I facets) or phase II. Larger teeth at a similar stage of wear will typically exhibit larger wear facets than smaller teeth. The area associated with each aspect of the power stroke, therefore, was expressed as a proportion of the total area of the wear facets across the occlusal surface. Wear facet inclination was measured in relation to the best-fit plane fitted to a curve drawn around the cervical margin of the tooth. This measurement is called the dip angle. Tip crushing areas, flat subcircular areas of wear on the cusp tips, have previously been attributed to the crushing action of the cusp tips in the opposing basins of the antagonistic teeth during puncture-crushing cycles [36, 39, 40]. These wear areas were, therefore, not considered in the current analysis which is concerned with wear facets primarily created with an inclination, indicating a horizontal directional component of jaw movement, during the power stroke. More details about conducting OFA are given in S1 Text.

Differences in wear facet area composition were visualised using a ternary plot. A ternary plot is a diagram that displays three variables which sum to a constant (1 or 100%). Each axis corresponds to one of the three variables that form the composition (buccal phase I, lingual phase I and phase II facet areas). Observations are plotted within the equilateral triangle formed by the three axes. The ratio of the three variables for a given observation are graphically displayed based on the position of the plotted point within the triangle and the proximity of the point to the apex associated with that element of the composition. Ternary diagrams were produced in R statistical software (v.3.6.1) using the package ‘compositions’.

**Fig 3:**
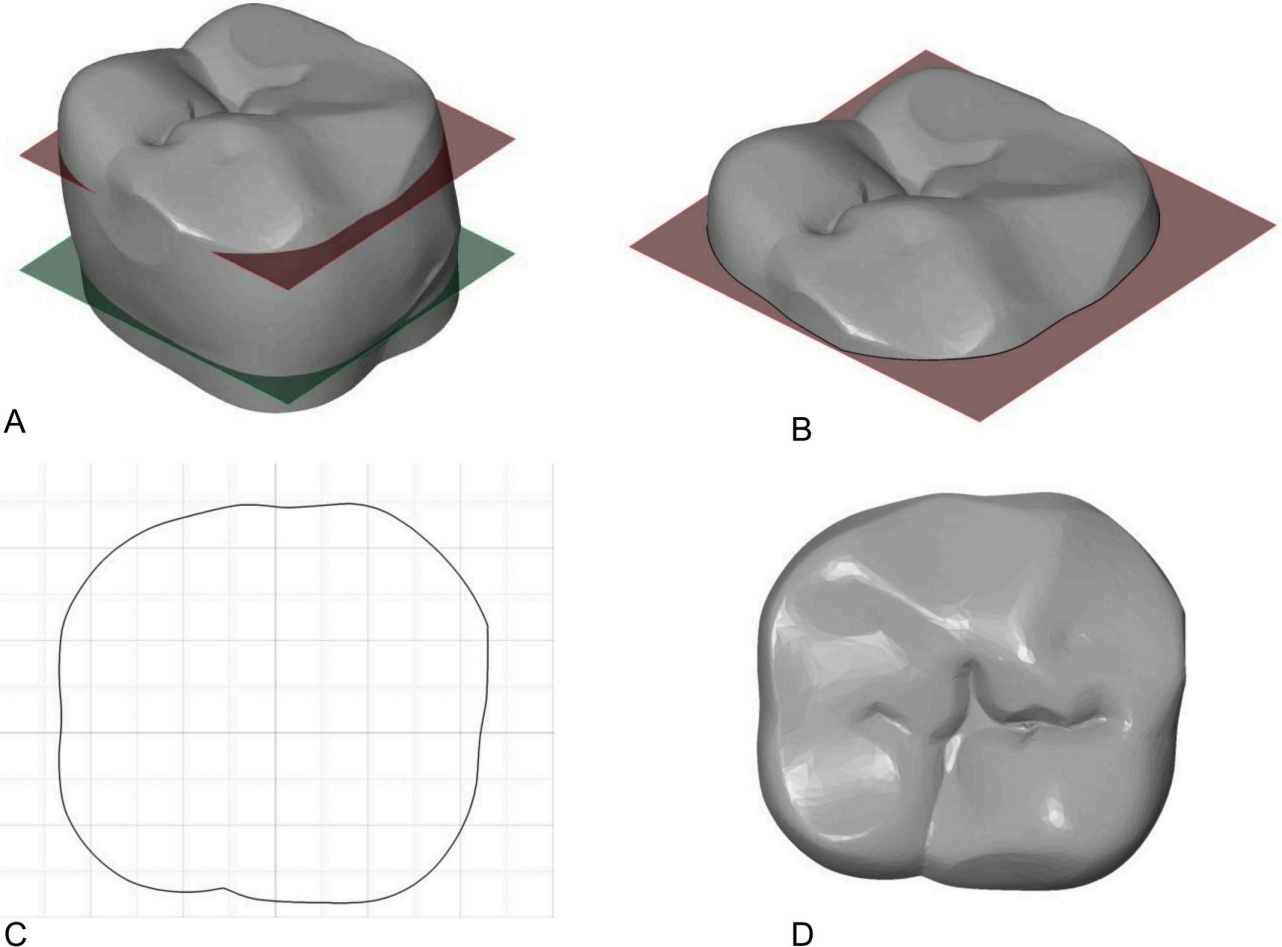
Process for calculating ORI using GOM Inspect. A) The cervical reference plane (green) is translated along the z-axis to the deepest point of the occlusal surface (red). This is typically the deepest point of the central fossa. B) The polygonal model is then cut at the level of the occlusal plane. C)The 2D area of the tooth is measured at the level of the occlusal plane. D) The 3D occlusal area is measured from the level of the occlusal plane. The occlusal relief index (ORI) is the area of D divided by the area of C.

In addition, the occlusal relief index (ORI) was calculated for each lower second molar, which provides a measure of the complexity and steepness of occlusal topography [70, 73, 74]. This value was calculated by dividing the 3D area of the occlusal topography above the level of the central fossa by the 2D area of the molar crown at the deepest point of the central fossa (Fig 3). The ORI values were compared between the industrial era and the medieval and early post-medieval periods using an independent sample t-test.

Kinematic simulations of the power stroke were conducted using the Occlusal Fingerprint Analyser software package developed by DFG Research Unit 771 (freely available at https://www.for771.uni-bonn.de/for771-en/ofa) on one individual from the medieval and early post-medieval periods and one from the industrial period. It provides a complementary approach to the static analysis of the molar wear pattern by indicating the directions of occlusal movements involved in wear facet development. The software enables the simulation of the occlusal phase of the power stroke between antagonistic teeth and can be used to infer the kinematic relationship between sets of wear facets. The user can set a trajectory pathway for the lower teeth which approximates their movement during the power stroke. The software detects collisions between the teeth as they come into contact and are deflected along the surface relief of each other. Contact areas can be recorded and assessed as they develop during the power stroke enabling comparisons to be made between individuals, groups and taxa. For further details on the operation of the Occlusal Fingerprint Analyser software refer to Kullmer *et al*. [30]. The OFA software data of these two individuals, alongside the data extracted from the wear facet patterns of the lower second molars of the whole sample, led to the development of theoretical models of power stroke behaviours for the pre-industrial and industrial groups.

### Statistical analyses

Wear facet composition in the two study groups was explored statistically using the methods developed by Aitchison [75] and implemented by Boogaart and Tolosana-Delgado [76] to overcome the issues with applying conventional statistical approaches to compositional data i.e. data in which each set of observations sum to 100%. Consequently, the data was subject to an isometric-log ratio transformation prior to further statistical analysis [77]. The isometric-log transformed data did not fulfil the assumption of multivariate normality, therefore, wear facet proportions in the industrial and pre-industrial groups were assessed using Permutational-MANOVA (PERMANOVA) applied to the Euclidean distance matrix of the data [78, 79]. The test compares the F-statistic generated using the data divided into pre-industrial and industrial groups with F-statistics values obtained by randomly exchanging observations between the two study groups (a permutation value of 9999 was selected). If there were no differences between the groups, the recorded observations would be exchangeable between the two groups without impacting the F-statistic value generated; the F-statistic generated using the actual data split between the two study groups would fall within the range of F-statistic values generated using the random permutations of the data. Statistical analysis was performed using the packages ‘RVAideMemoire 0.9–66’ and ‘compositions’ in R statistical software (refer to S1 Text for code used). The PERMANOVA procedure outlined above was also used to test the effect of age-at-death and sex on wear facet expression in the two groups.

The mean dip angle for each wear facet type (buccal phase I, lingual phase I and phase II) was calculated for each lower second molar examined. Wear facet dip angle values satisfied the assumptions of normality and homogeneity of variance (Shapiro-Wilk test p>0.05; Levene’s Test p>0.05), therefore, differences in dip angle inclination between the two human groups were tested using the independent sample t-tests. Independent sample t-tests were also used to determine the effect of sex and age-at-death on wear facet dip angle in each of the human groups.

## Results

### Static OFA

**Fig 4:**
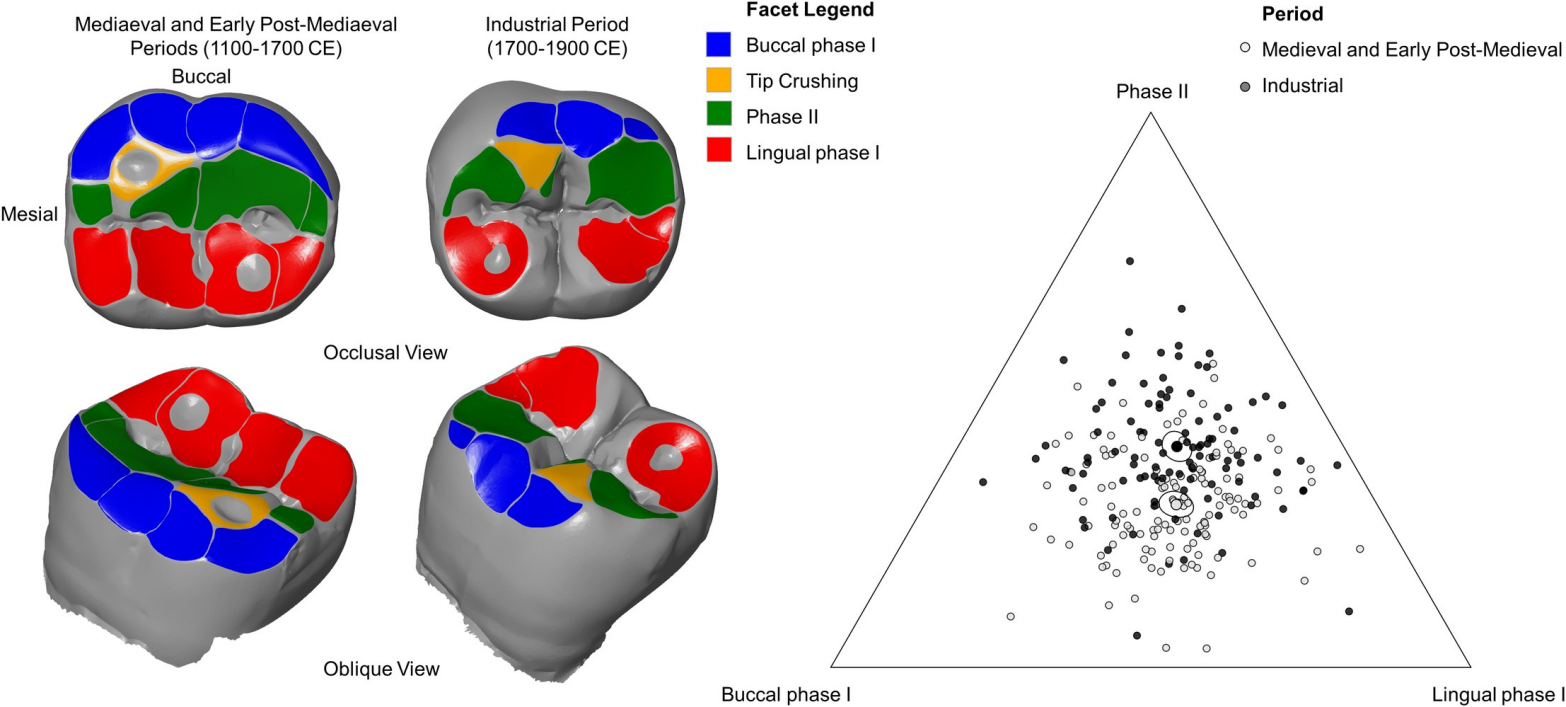
Comparison of dental wear facet patterns between the pre-industrial and industrial groups. Above: Comparison of lower second molar dental wear facet patterns between the medieval and early post-medieval Periods and the industrial Period. Wear facets are marked as follows: buccal phase I facets are blue, lingual phase I facets are red, phase II facets are green and tip crushing areas are orange. Note large phase II wear facets in the industrial period relative to buccal phase I wear. In the earlier periods, lingual phase I wear facets and buccal phase I facets occupy a larger proportion of the overall wear facet area. Below: Ternary plot showing the relationship between period and relative wear facet area of the lower second molar (composed of buccal phase I (BPI), lingual phase I (LPI) and phase II (PII) facet areas). The industrial group is displayed as dark grey circles and the medieval and early post-medieval group as light grey circles. The centre value for each period is represented as a larger filled shape surrounded by 95% confidence regions. The industrial group is represented by a large black circle and the medieval group a white circle.

The wear facet area composition in the molars differs significantly between the two groups (Permutational Multivariate analysis of variance p<0.05; Fig 4 and Table 4). In the medieval and early post-medieval group, buccal phase I and lingual phase I wear facet areas occupy a larger proportion of the total wear facet area than phase II wear facets (mean composition: buccal phase I 30.88%, lingual phase I 39.84% and phase II 29.27%). The industrial assemblages possess greater proportions of phase II wear as a consequence of reduced phase I wear regions (Mean: buccal phase I 26.25%, lingual phase I 34.05% and phase II 39.69%) (Fig 3). The variance in dental wear facet area proportions does not differ significantly between the two periods (Permutational homogeneity of multivariate dispersion test p>0.05; Table 5; Standard Deviation: industrial period = 0.45; medieval and early post-medieval Period = 0.40).

**Table 4:** Results of the one-way permutational multivariate analysis of variance (PERMANOVA) assessment of the relationship between period and lower second molar wear facet area composition. The dependent variables being tested are buccal phase I, lingual phase I and phase II wear facet area and the independent variable is the two modern human groups (either pre-industrial or industrial).

Factor	Degrees of Freedom	Sum of Squares	Mean of Squares	F-model	R^2^	p-value	Null Hypothesis
**pre-Industrial vs Industrial**	1	8.56	8.56	19.46	0.08	0.0001	Rejected
**Residuals**	231	101.62	0.44		0.92		
**Totals**	232	110.19			1.00		

Permutation value was set at 9999. Null hypothesis: Wear facet area composition did not significantly differ between the pre-industrial and industrial groups.

**Table 5:** Results of permutational test assessing homogeneity of multivariate dispersions for comparison of wear facet area composition between the pre-industrial and industrial groups.

	Degrees of Freedom	Sum of Squares	Mean of Squares	F-model	p-value	Null Hypothesis
**Pre-Industrial vs. Industrial Group**	1	0.23	0.23	1.63	0.21	Not Rejected
**Residuals**	231	33.24	0.14			

Null hypothesis: within group variation did not differ significantly between the two periods. The null hypothesis of homogeneity of dispersion is supported by p>0.05.

No significant differences were found in wear facet area composition between the cemetery assemblages within the pre-industrial and industrial groups following post-hoc analysis (Table 6). When comparing the industrial and pre-industrial cemetery assemblages, a significant difference was apparent between the Hereford Cathedral assemblage and the industrial-era St Bride’s and St Michael’s Litten material. The St. Bride’s material also differed significantly from the York Barbican, Box Lane and St James’ leprosarium assemblages. The industrial portion of the St Michael’s Litten, Chichester, assemblage differed significantly from the York Barbican, Box Lane and St James’ leprosarium, Chichester, material.

**Table 6:** Results of post-hoc pairwise comparison using permutational MANOVA of wear facet area between the skeletal assemblages examined.

	York	Blackfriars	Box Lane	St James, Chichester	Coronation Street	St Michael’s Litten (pre-industrial)	St Michael’s Litten (industrial)	Hereford Cathedral	St Bride’s
Blackfriars	0.09	NA	NA	NA	NA	NA	NA	NA	NA
Box Lane	0.28	0.09	NA	NA	NA	NA	NA	NA	NA
St James, Chichester	0.84	0.10	0.11	NA	NA	NA	NA	NA	NA
Coronation Street	0.09	0.84	0.10	0.11	NA	NA	NA	NA	NA
St Michael’s Litten (pre-industrial	0.10	0.84	0.06	0.12	0.89	NA	NA	NA	NA
St Michael’s Litten (industrial	**0.00**	0.09	**0.00**	**0.00**	0.12	0.09	NA	NA	NA
Hereford Cathedral	0.61	0.28	0.61	0.89	0.11	0.28	**0.00**	NA	NA
St Bride’s	**0.00**	0.30	**0.00**	**0.00**	0.61	0.28	0.28	**0.00**	NA
St Peter’s	0.09	0.54	**0.02**	0.09	0.89	0.77	0.21	0.13	0.61

Permutation value was set at 9999.

**Fig 5:**
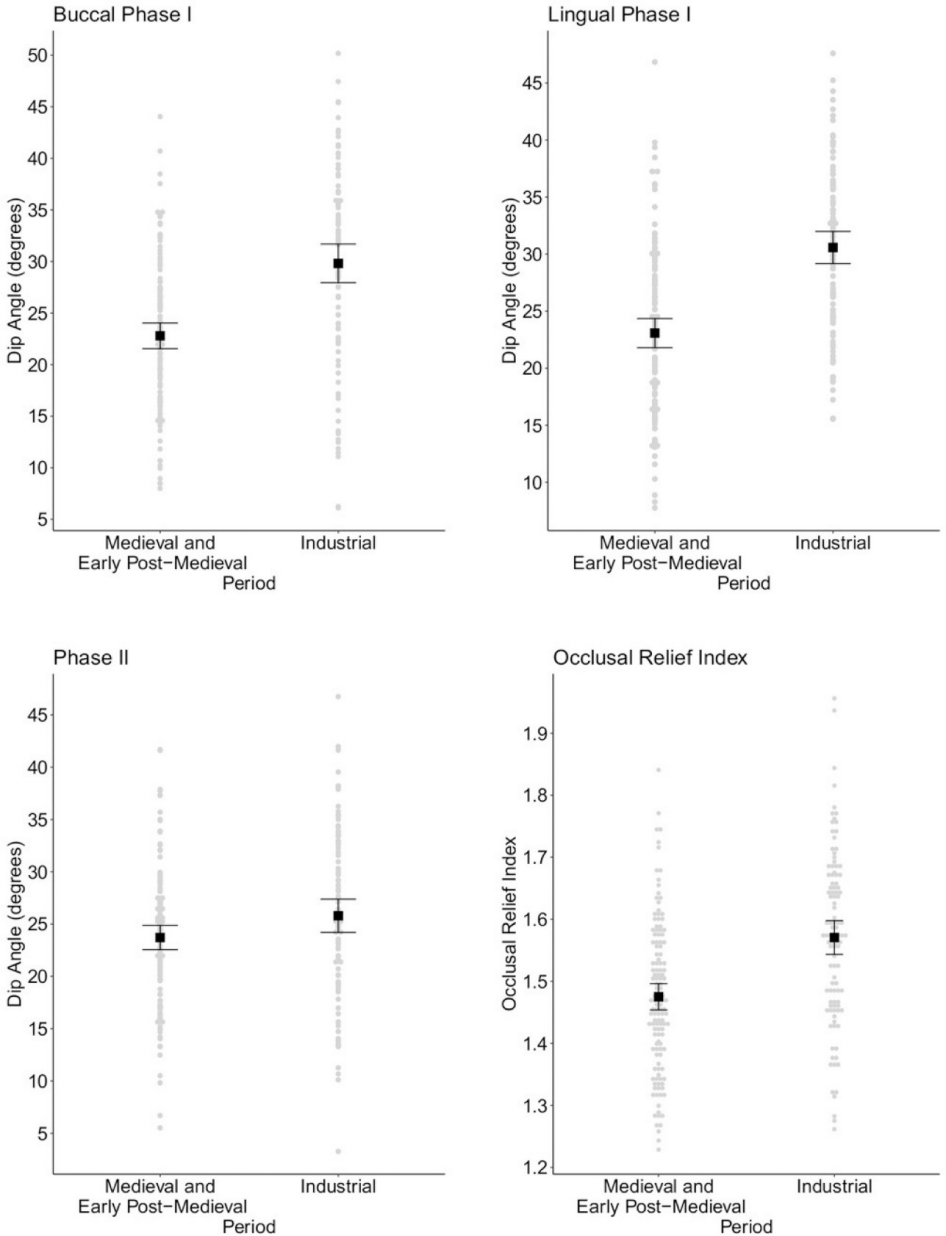
Comparison of dip angle and ORI between the pre-industrial and industrial group. Upper row and lower left: Plots comparing mean dip angle value for the lower second molars for buccal phase I, lingual phase I and phase II facets between the medieval and early post-medieval periods and the industrial period. The means are represented by black squares alongside error bars giving 95% confidence intervals. Grey dots visualise the dip angle value for each of the lower second molars assessed using OFA. Lower right: Plot comparing mean Occlusal Relief Index values for the lower second molars between the two periods (black squares with 95% confidence intervals). Grey dots visualise each individual ORI value for the lower second molar targeted by OFA.

Wear facet dip angles of the lower second molar also differ significantly between the pre-industrial and industrial groups. Buccal phase I and lingual phase I mean dip angles are significantly steeper in the industrial assemblages (Independent sample t-test p<0.05; Fig 5 and Table 7). Phase II wear facets are as expected also more steeply inclined in the industrial period. This difference approached significance; however, the null hypothesis could not be rejected for phase II dip angles as the 95% confidence interval includes the p-value (Table 7).

**Table 7:** Result of independent sample t-tests comparing mean dip angles of the wear facets of the lower second molar between the industrial and pre-industrial groups.

Power Stroke Phase	Buccal Phase I	Lingual Phase I	Phase II
Industrial Mean Dip Angle(°)	29.81	30.58	25.79
Industrial Standard Deviation	9.59	7.22	8.08
Medieval Mean Dip Angle (°)	22.79	23.08	23.71
Medieval Standard Deviation	7.18	7.35	6.72
t-value	6.19	7.8	2.09
Degrees of Freedom	183.97	220.69	195.23
p value^a^	<0.001	<0.001	0.038
Effect Size	0.84	1.03	0.28
95% CI Effect Size	0.57 to 1.11	0.75 to 1.30	0.02 to 0.55
Statistical Power	1.00	1.00	0.85
Null Hypothesis	Rejected	Rejected	Not Rejected

^a^ Bonferroni adjusted p-value for 3 tests = 0.017.

Dip angle data was normally distributed (Shapiro Wilk test BPI p-value = 0.29; LPI p-value = 0.15; PII p-value = 0.64). Null hypothesis: The dip angle values for the wear facets associated with a given phase of the power stroke did not differ significantly between the two periods.

Significantly greater occlusal relief index (ORI) values were observed in the lower second molars of individuals dating to the industrial period (Independent sample t-test p<0.05; Fig 5 and Table 8). This indicates that higher relief and topographic complexity across the occlusal surface characterise the industrial group.

**Table 8:** Result of independent sample t-test examining the effect of period on Occlusal Relief Index (ORI) of the lower second molar.

Industrial Mean Occlusal Relief Index	1.57
Standard Deviation	0.14
Medieval Mean Occlusal Relief Index	1.48
Standard Deviation	0.12
t-value	5.48
Degrees of Freedom	206.12
p-value	<0.0001
Effect size	0.73
95% CI effect size	0.46 to 1.00
Statistical power	1.00
Null Hypothesis	Rejected

The data was normally distributed (Shapiro-Wilk test W = 0.99, p = 0.08) and homogeneity of variance could be assumed (Levene’s test F value = 1.72, p = 0.19). Null hypothesis: ORI did not differ significantly between the two periods.

Wear facet expression was not found to differ significantly between males and females in either the pre-industrial or industrial group (S1–S4 Tables in S1 Text).

Age-at-death category had a significant effect upon wear facet composition in the industrial group. The associated R^2^ was of a smaller magnitude than that obtained when examining the period-based influence on wear facet area composition, however (0.05 and 0.09, respectively) (Table 9). The older age-at-death group had larger proportions of Lingual Phase I wear facets and smaller proportions of Buccal Phase I and Phase II wear when compared to the younger age-at-death category. The centre values for the younger age category were BPI 28.69%, LPI 31.13% and PII 40.17%. Centre values for the older age category were BPI 21.84%, LPI 42.21% and PII 35.95%. Age-at-death could not be explored in the pre-industrial assemblage as the older age-at-death category was poorly represented in the material examined.

**Table 9:** Results of Type I PERMANOVA assessing the relationship between age-at-death category (either younger or older) and wear facet area composition in the industrial material.

	Df	Sum of Squares	Mean of Squares	F-model	R^2^	p-value	H_0_
**Age-at-death Category**	1	2.23	2.23	3.76	0.06	0.027	**Rejected**
**Residuals**	57	33.80	0.59		0.94		
**Total**	58	36.03			1.00		

Null hypothesis: wear facet area composition did not differ significantly between the younger and older age-at-death categories dating to the industrial period.

BPI dip angles were significantly less steep in the older age-at-death category (Table 10). LPI and PII wear facets were slightly more shallowly inclined in the older age-at-death category. The effect size of age-at-death on BPI wear facet dip angle was of a smaller magnitude than the effect size associated with period (maximum -0.50 and 0.84, respectively).

**Table 10:** Independent sample t-tests assessing age-related differences in dip angle during the industrial period for BPI, LPI and PII wear facets.

Wear Facet Function	BPI	LPI	PII
**Younger Mean (°)**	31.38	31.52	26.94
**Standard Deviation**	8.34	7.52	8.09
**Older Mean (°)**	26.78	28.92	24.8
**Standard Deviation**	9.76	6.8	8.86
**t-value**	-2.31	-1.75	-1.18
**Degrees of Freedom**	73.39	82.27	68.01
**p value** *	0.02	0.08	0.24
**Effect Size**	-0.5	-0.36	-0.26
**95% CI Effect Size**	-0.92 to-0.07	-0.78 to -0.6	-0.68 to 0.16
**Statistical Power**	0.63	0.38	0.22
**H**_**0**_	Rejected?	Not Rejected	Not Rejected

*Bonferroni adjusted p-value = 0.017. The data for each variable was normally distributed (Shapiro-Wilk p>0.05) and exhibited homogeneity of variance (Levene’s test p>0.05). Null hypothesis: dip angle values did not differ significantly between younger and older age-at-death categories during the industrial period for each of the wear facet types assessed.

### Dynamic OFA

**Fig 6:**
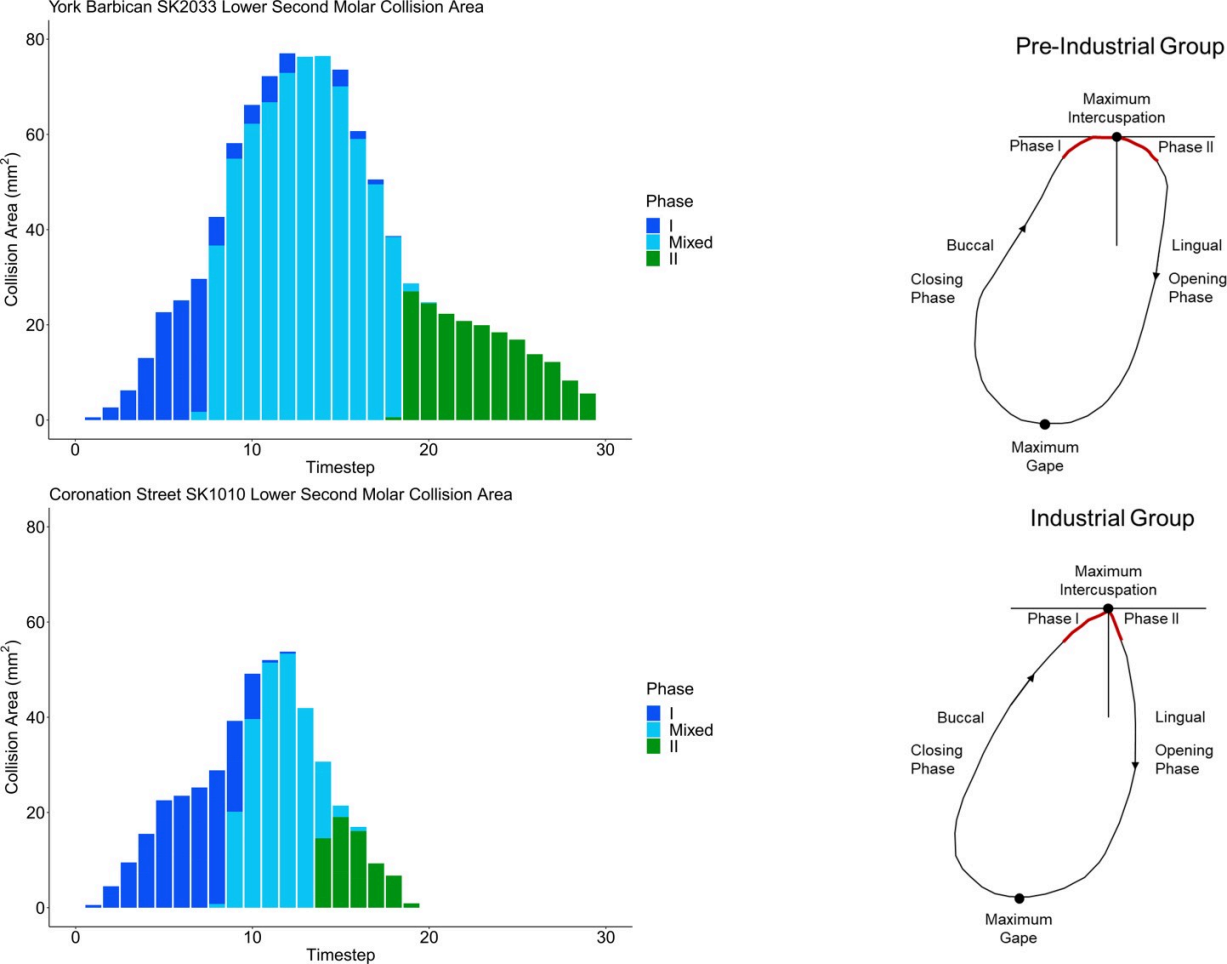
Development of contact areas during power stroke simulations and proposed chewing models. Left: Stacked bar charts comparing the development of wear facet areas for power stroke simulations conducted using the Occlusal Fingerprint Analyser software package. Each collision is broken down into a series of timesteps in which the lower tooth model attempted to move 0.05mm towards the next point along the estimated power stroke movement trajectory. The software calculates any collisions that occur between the opposing teeth at each time step. One individual was selected from the medieval period (SK2033 from York Barbican) and one individual from the industrial period (SK1010 from Coronation Street). The medieval individual showed an extended power stroke relative to the industrial individual and a more clearly defined and extended period of exclusively phase II contacts. Right: Visualisation of the hypothesized differences between the chewing cycles of individuals from the industrial and pre-industrial group.

The molars in the pre-industrial group performed a greater lateral displacement during phase I of the power stroke resulting in a more gradual increase in crown contact area relative to the industrial period (Fig 6 and S1 and S2 Videos). The phase II movement of the pre-industrial individual took place over an enlarged time segment and involved greater medial displacement of the lower molars when compared to the industrial individual. The industrial-era individual was characterised overall by smaller and steeper antagonistic contact areas between molars during a shortened power stroke sequence when compared to the individual from the pre-industrial group.

## Discussion

### Dental macrowear patterns in the medieval and early post-medieval periods and the Industrial Revolution

The study aimed to assess whether the dental occlusal pattern typical of individuals from the Industrial Revolution indicated changes in the occlusal power stroke when compared to their medieval antecedents. This was framed in terms of the dietary changes that took place during the Industrial Revolution, which were inferred from archaeological, historical and bioarchaeological evidence. Nine British cemetery assemblages were included and compared. Evidence for changes in the power stroke were inferred from the dental wear facet patterns of the lower second molars using OFA. Significant differences were found between the dental wear facet patterns of individuals dating from the industrial era and those from the medieval and early post-medieval periods.

The dental macrowear patterns of the lower second molars of the pre-industrial group are characterised by significantly greater proportions of buccal phase I and lingual phase I wear when compared to the industrial group. This indicates differences in chewing behaviours between the two groups. A greater transverse jaw movement has been associated with the enlargement of lingual phase I wear facet areas in some modern hunter-gatherers [40]. This included groups, such as the Khoe-San and Australian Aborigines, who were more reliant on hard and abrasive food stuffs, such as seeds, but also tough plant parts [38, 40]. Larger buccal phase I wear facet areas in the pre-industrial group are consistent with a prominent and prolonged shearing action during the incursive portion of the masticatory stroke [36, 40]. In contrast, industrial individuals frequently exhibit more poorly developed phase I wear facets alongside wear facet patterns dominated by phase II wear. This occlusal situation reflects a briefer and more vertically directed phase I shearing action, such as designated by small and steep wear facets, and consistent with the consumption of less abrasive and softer food items that do not require extensive transverse jaw movements during oral processing [39, 80–82]. Similarly, the OFA kinematic simulations for each period further indicate a reduction in lateral travel of the lower teeth during the power stroke in the industrial period. Previously assumed differences in the physical properties of the dietary staples consumed in each period are confirmed by the differences in dental wear facet patterns observed in the current study [14].

The variance in dental wear facet patterns did not differ significantly between the two periods. Nevertheless, variance values are relatively large in both. This might derive from the inclusion of individuals of both higher and lower social status within the pre-industrial and industrial samples. In the medieval period, the quality and type of bread consumed varied between social classes. The upper classes ate fine wheaten bread while the lower classes would consume darker and coarser loaves of rye, barley and oats [43]. For the peasantry, dietary supplements, such as meat, would have formed as little as 20% of the total calories consumed. This was limited to salted meat and bacon, which were more readily available in the autumn once livestock were fattened otherwise dairy products acted as the ‘white meat’ of the poor [83]. In contrast, the diets of the upper classes were supplemented daily by either meat or fish. [84]. In the industrial period, the quantity of meat eaten remained a crucial indicator of social status with the poorest subsisting on only bread, cheese and potatoes [46]. The middle and upper classes would often consume meat daily [7]. The contextual information available for most of the skeletal assemblages examined was not adequate to determine whether social status influenced the variability in the dental wear facet patterns observed in either period. Adequate contextual information pertaining to social status was available, however, for the St Michael’s Litten and St Bride’s assemblages. The relationship between social status and dental wear patterns in these assemblages has been considered in Silvester [60].

The increasingly steep wear facets and greater occlusal relief in the molars of the industrial group reflects a reduction in the transverse component of the masticatory stroke as well as a reduction in abrasive particles within the food eaten [38, 62, 85]. This is consistent with the inferences made using the wear facet area data. The steeper inclination of dip angles in the industrial period points to a reduction in the demands placed on the masticatory system as dietary content became less hard, but also less tough, as a consequence of more intensive food processing. Similarly, Smith [62] argued that more oblique wear in agriculturalist groups relative to hunter-gatherers was closely associated with their consumption of more heavily processed and cooked grains. In addition, clinical feeding studies have found that tougher foods are more frequently chewed with a larger lateral component of jaw movement, whereas, soft food, such as modern bread, are characterised by slim drop shaped movements often with a greater vertical amplitude, when jaw movement is viewed in the frontal plane [86–88]. It can be hypothesized, therefore, that the extended lateral shift that characterised the chewing action of the pre-industrial group resulted in greater flattening of the occlusal relief which was further accelerated by the inclusion of greater quantities of abrasive material within the diet. Further shifts in food processing technology have resulted in the retention of virtually unworn teeth late into life among many industrialised groups in the 21^st^ century [3, 89–91].

### Additional factors that may have influenced wear facet expression and limitations of the current study

No significant differences in dental wear facet expression were found between the sexes in the industrial period. The bulk of the dietary staples consumed in the 18th-19th centuries would have been consistent between the sexes. Both sexes would have derived most of their calories from bread, potatoes, vegetables and sweetened tea. Men, within a lower-class urban setting, however, would receive the majority of meat that could be afforded. Even so, the quantity would likely be meagre and would therefore be less likely to impact habitual masticatory behaviours [7, 43, 92]. Similarly, historical accounts indicate that sexual differences in the diets consumed in the medieval and early post-medieval periods were typically limited to supplementary items [84].

In the industrial period, individuals estimated to be older exhibited slightly greater proportions of lingual phase I wear. Clinical research into the effect of ageing on chewing cycles have found that older individuals still retain the capacity to adapt to changing food properties, including food hardness [93–96]. In addition, there was little historical evidence to support dramatic shifts in the physical properties of the foods eaten in advanced age in either period [7, 8, 97]. Consequently, largely consistent responses in masticatory parameters would be anticipated to the softer and more heavily processed foodstuffs consumed in the industrial period irrespective of age. A smaller R^2^ value was associated with the differences in wear facet proportions between age-at-death categories in the industrial period when compared to the R^2^ value associated with the differences in wear facet proportions between the two periods (Tables 4 and 9). As a result, the different age distributions of the pre-industrial and industrial groups compared should not have markedly impacted the results of the overall comparison between the pre-industrial and industrial groups.

The older portion of the industrial assemblage typically exhibited greater cusp reduction and less obliquely inclined phase I wear facets. As wear advances with increasing age, further modification of the power stroke is predicted in order to maintain functional masticatory efficiency as occlusal topography provides less of a guiding function [37, 98]. An increase in the lateral portion of the power stroke might be anticipated with increased age based on observations of modern hunter-gatherers with extremely advanced tooth wear [3], but, a broader range of occlusal wear stages and skeletal age-at-death categories would be required to test this hypothesis further.

The individuals examined in the current research only represented a small cross-section of the potential age-at-death ranges within each assemblage due to the restrictive selection criteria required for the performance of wear facet analysis. OFA can only be effectively applied to lightly worn teeth [39, 48] and is largely inappropriate for the analysis of wear following the obliteration of dental wear facets after which only an assessment of the overall inclination of the wear plane of the tooth can be made. Faster wear rates meant that only relatively young individuals could be included from the medieval and early post-medieval periods, to ensure that wear stages of the lower second molars were comparable between the two periods. For this reason, the influence of age-at-death on wear expression could not be assessed in the pre-industrial group.

The current project focused on the occlusal wear facet pattern of the lower second molar. This decision was driven by work on primates, which has found that the lower second molar provides an effective representation of masticatory function within one species [62, 99]. In addition, the first molars were more commonly lost ante-mortem in the assemblages examined, particularly those dating to the industrial period. Wear facet expression has been previously shown to differ significantly between the first and second molars in modern hunter-gatherers [100]. This is likely due to functional differences between the molars from anterior to posterior, which relate to subtle differences in dental morphology and their position and inclination in relation to the TMJ [101]. Thus, the wear facet data derived from the current project can only be directly compared with other research examining lower second molars [e.g., 41].

Wear facet expression may have been influenced by additional confounding factors which could not be fully considered as they were beyond the scope of the project. The presence of occlusal variability can modify masticatory patterns and even restrict the pathways of movement of the mandible that are possible during chewing [102]. The inclination of the molar teeth in the jaws may also influence the size and inclination of the wear facets that develop due to differences in how the teeth occlude during the power stroke [103]. Bruxism involving the repetitive grinding and clenching of the teeth can also modify dental wear facet patterns [104]. Tooth wear alone cannot be used to diagnose bruxism within a bioarchaeological context and an appropriate methodology to do so requires further development [105]. Clinical dental research links bruxism with psychosocial stress [106]. Dramatic changes did occur in the social environment during the industrial era [7], but it is difficult to quantify whether levels of psychosocial stress increased relative to the medieval period [107].

### The role of phase II wear facets in the power stroke

The large proportion of phase II wear (>40%) in individuals from the industrial period contrast with previous studies that have examined wear facet area proportions in modern hunter-gatherers and Palaeolithic anatomically modern humans and Neanderthals [38, 40] and the majority of pre-industrial individuals examined in the current analysis. The role and mode of formation of phase II wear facets during the power stroke remains debated [108, 109]. In extant ungulates, large phase II facets have been associated with a more heavily frugivorous diet [36]. This relationship between increased frugivory and large phase II wear facets was not apparent in modern hunter-gatherers [40]. Large phase II wear facets may indicate extended excursive contacts during the power stroke, more consistent with the larger central fossa present in great ape lower molars rather than anatomically modern humans [41, 62]. This indicates that phase II facet areas in industrialising modern humans are likely principally performing a different role during mastication.

Some authors have associated phase II facets with a grinding function, combining a flat-angled shearing and crushing action, during phase II of the power stroke [28, 36]. Experimental observation of bone strain and muscle activity during the power stroke in primates and dental microwear textures, however, indicated that the forces principally responsible for phase II facet creation are likely active towards the end of phase I, immediately prior to, and possibly during, maximum intercuspation [108–110]. Similarly, the peak in contact force also occurred during maximum intercuspation during a finite element analysis simulation involving antagonist human first molars and their supporting tissues [111]. A similar pattern of jaw adductor muscle activity was found in a study of EMG profiles during mastication in modern humans. Peak muscle activity occurred towards the end of phase I just prior to maximum intercuspation and declined rapidly during jaw opening [112]. Phase II wear facets were also involved immediately prior to maximum intercuspation during the power stroke simulations conducted for the current project. As a result, a dramatic increase in wear facet contact area occurred in the individuals examined during the terminal portion of phase I of the power stroke (Video 1 and 2). This period of mixed phase I and II contacts corresponds with the peak in jaw adductor muscle activity described in EMG studies of modern humans and other non-human primates [109, 111, 112].

In this model of food breakdown during phase I of the power stroke, material is cut initially along the edges of phase I wear facets. In addition, food particles are compressed against phase II wear facets, formed along the lingual slopes of the buccal cusps far down into central fossa of the lower molars, during the terminal part of phase I and during maximum intercuspation, proximate to or during the peak in jaw adductor muscle force. The extensive development of phase II wear facets when compared to the size of phase I wear facets in the industrial-era may, therefore, indicate that food breakdown during the power stroke may principally have occurred as a result of the crushing action against phase II facets during the terminal part of phase I.

## Conclusions

Dentists often desire predictable and stable outcomes for their patients during and following treatment [113]. Dental practitioners need to be aware of the functional consequences changes in food properties have had on the masticatory system and craniofacial development over the past three centuries. The current research highlights the biomechanical feedback loop that exists between food properties, chewing behaviours and dental wear. The differences interpreted in masticatory power stroke between the pre-industrial and industrial groups in the current study likely underlay the developments in occlusion and craniofacial morphology that have been previously reported as a result of the dietary changes that accompanied industrialisation [2, 14]. A reduction in the lateral component of jaw movement and chewing duration during and following the Industrial Revolution due to the consumption of an increasingly soft diet diminished the demands placed on the craniofacial complex during mastication [3, 15, 16]. This may fail to promote craniofacial growth and development to the extent that the hard and tough ancestral diet of humans did resulting in changes in jaw morphology [14, 114, 115] and lead to an increase in malocclusion over time [1, 2]. The changes seen in the industrial era sample in this study reflect the impact of the early stages of the industrialisation of diet on occlusal wear patterns and chewing kinematics, and may reflect the beginning of contemporary dental behaviours.

As food properties are modified further in the future by both technological changes and social factors, more frequent and perhaps more marked disturbances related to dental occlusion might be anticipated. Diet in the 21^st^ century have moved increasingly towards ultra-processed foods; they form the principal source of dietary calories in the USA and UK [116–118]. These foods are mostly industrial formulations, made from substances extracted from foods alongside additives, which are designed to be hyper-palatable and ready-to-consume. They are also often soft, quick to eat and undemanding to chew [119, 120]. An increase in the prevalence of diet related diseases has been attributed to the growing reliance on these foods due to their high levels of unhealthy types of fat, refined starches, free sugars and salt [10]. Little consideration has yet been made of their impact on the growth and development of the masticatory system. Studies have reported secular trends in the increased prevalence and severity of malocclusion over the past century, which may reflect the greater uptake of these heavily processed foods [121–123]. As a consequence, clinicians may need to be active in the physical training of the masticatory system to ensure more optimal development in the absence of appropriate dietary stimuli. Otherwise, negative functional consequences may result if the masticatory system is not able to adapt to the rapid changes in food properties that are currently taking place.

## Supporting information

S1 Text (DOCX)

S1 Video Visualization of the power stroke simulation of individual SK2033 dated to the medieval period performed using the Occlusal Fingerprint Analyser software package. The movement trajectory of the lower teeth is more shallowly inclined and begins from a more laterally shifted position during phase I of the power stroke when compared to the industrial individual. Phase II occurs over a more elongated time segment and involved greater medial movement of the lower teeth.
(MP4)

S2 Video Visualization of the power stroke simulation of individual SK1010 dated to the industrial period (AD 1700–1900) performed using the Occlusal Fingerprint Analyser software package. This simulation showcases the movement trajectory of the lower teeth during the power stroke. Note an oblique angle of approach during phase I of the power stroke and a very brief phase II movement following maximum intercuspation.
(MP4)

S1 File (PDF)

S1 Data (CSV)
