# Supplementary material for: A dental revolution: The association between occlusion and chewing behaviour
Source: PLoS One. 2021 Dec 15;16(12):e0261404. doi: 10.1371/journal.pone.0261404 (PMC8673603; doi:10.1371/journal.pone.0261404)
Supplement: S1 Text — (DOCX) [file pone.0261404.s001.docx]

**S1 text for:**

A dental revolution: the association between chewing behaviours and malocclusion.

Authors:

Christopher Martin Silvester^1^

Ottmar Kullmer^23^

Simon Hillson^1^

**Author Affiliations:**

1 Institute of Archaeology, University College London, London, WC1H 0PY, United Kingdom

2 Department of Paleoanthropology, Senckenberg Research Institute and Natural History Museum Frankfurt, 60325 Frankfurt, Germany

3 Department of Paleobiology and Environment, Goethe University, 60439 Frankfurt, Germany

**Corresponding Author:**

**Email:**  [christopher.silvester.15@ucl.ac.uk](mailto:christopher.silvester.15@ucl.ac.uk)

**Methods: occlusal fingerprint analysis (OFA)**

Previous studies [e.g. 1, 2] have developed and utilized a workflow for conducting OFA in the software package Polyworks (Innovmetric). This software is prohibitively expensive to many researchers. As such, the present study developed a method for performing OFA in GOM Inspect (GOM, Braunschweig, Germany) which was identified as a freely available alternative. The principles underlying OFA have been detailed recently [3)], therefore, only the steps involved in conducting the method will be dealt with here.

The workflow detailed was performed using GOM Inspect Version 2018 Hotfix 6. Firstly, the 3D model of the lower second molar mesh was prepared for analysis. In virtual models involving multiple teeth, the lower second molar was first isolated by selecting and deleting the adjacent teeth. Occasionally very small air bubbles were present on the dental gypsum models and these defects were reproduced on the surface of the virtual dental model created by the structured light scanning system (GOM ATOS 80 scanner, GOM, Braunschweig, Germany). These areas were manually identified, outlined and removed from the virtual model and the surface reconstructed using the close hole function in GOM Inspect. Teeth cut from molar tooth rows were frequently missing mesial and distal surfaces obscured by neighboring teeth. Following segmentation from the molar row, any missing surfaces were virtually reconstructed in GOM Inspect providing an approximation of the actual surface. This process utilized the existing 3D mesh to extrapolate the geometry of the missing 3D surface by building a series of mesh bridges across the missing area. The spaces between the bridges were then filled using the close hole function. Following this, the tooth was ready for further analysis.

OFA requires each specimen to be orientated in the same way prior to wear facet analysis. This was achieved using a standard reference plane fitted to the cervical margin of the tooth. A curve was drawn around the cervix of the tooth and the area 0.2mm either side of it was selected. A gaussian best-fit plane was then fitted to the selected area (Fig 2). The reference plane needs to be independent of the occlusal surface as dental wear progressively modifies and obliterates landmarks on the crown [1, 4]. The cervical plane of each tooth model was then translated to a standardized x-y plane. The buccal direction was aligned with the positive y direction, the mesial axis of the tooth was aligned with the positive x direction (in left teeth) and the occlusal direction was aligned with the positive z direction (Fig 2).

Once aligned, the wear facets on the occlusal surface of each specimen were delineated using surface curves. The mesh area bounded by the curve demarcating the border of each wear facet was selected in turn and measured to give the area of the wear facet in mm^2^ (Fig 2). For each tooth, wear facets were first grouped according to their functional role during the power stroke: buccal phase I wear (facets 1, 2, 3 and 4), lingual phase I wear (facets 5, 6, 7 and 8) and phase II wear (facets 9, 10, 11, 12 and 13). The relative proportion of the total wear facet area constituted by each wear facet group was then calculated. Tip crushing areas were also identified predominantly on the tips of the buccal molar cusps. These wear areas lack a clearly defined dip direction, are frequently more shallowly inclined than wear facets associated with the power stroke and have more poorly demarcated boundaries [1]. They cannot be definitively attributed to the normal power stroke and may be principally active during puncture-crushing cycles so were not considered in the current analysis [5, 6]. Wear facet inclination was measured in relation to the reference plane created using the cervical margin of the tooth. A gaussian best-fit plane was fitted to each wear facet and the angle between this plane and the reference plane was measured to give the dip angle for each wear facet (Fig 2).

Occlusal Relief Index (ORI) was calculated for each lower second molar. This value describes the steepness and surface complexity of the occlusal topography with larger values indicating a steeper and more complex surface [7]. This value is calculated by dividing the 3D area of the occlusal topography above the level of the central fossae by the 2D area of the molar crown at the level of the central fossae (Fig 4). In GOM Inspect, the cervical reference plane was first translated along the z-axis to the level of the central fossa. A section was then created through the tooth model at this level to create an occlusal plane parallel to the cervical reference plane. The cross-sectional area of the lower second molar at the level of the occlusal plane could then be measured in mm^2^. The 3D area of the occlusal surface above the occlusal plane was then also measured in mm^2^. The 3D area of the crown above the occlusal plane was then divided by the 2D area of the crown at the level of the occlusal plane to give the occlusal relief index.

**Statistical analysis: R code used for performing MANOVA**

#load packages

library(compositions)

library(RVAideMemoire)

library(vegan)

#get data

WAF<-read.csv("DentalRevolution_OFAdata.csv", header=T, na.strings=c("","NA"))

#conduct isometric log transformation of wear facet composition data

WAclo<-clo(WAF,parts=c("BPI","LPI","PII"),total=100)

WA<-acomp(WAclo)

#load dependent variables

Period<-WAF$Period

Period<-factor(Period, levels=c("pre-Industrial", "Industrial"))

#Calculate base for isometric log ratio transformation. This creates a dendrogram from which the base can be constructed for the wear facet composition data (see [8] for theoretical background).

dd = dist(t(clr(WA)))

hc1 = hclust(dd,method="ward.D")

plot(hc1)

mergetree=hc1$merge

CoDaDendrogram(X=acomp(WA),mergetree=mergetree,col="grey49",range=c(-8,8),box.space=1)

V<-balanceBase(WA,~((PII/LPI)/BPI))

#Create the isometric log-transformed data object.

WAilr<-ilr(WA,V=V)

#Create the distance matrix using the euclidean distance method.

Yperiod2<-vegdist(WAilr, method="euclidean", binary=FALSE, diag=FALSE)

#code for performing MANOVA

WearAreavsPeriodcomp<-adonis(Yperiod2~WAF2$Period,permutations=9999)

#homogeniety of dispersion test for wear area vs period.

mod2<-betadisper(Yperiod2,WAF$Period, type = c("median"), bias.adjust = FALSE, sqrt.dist = FALSE, add = FALSE)

## Permutation test for F

disper_PERMANOVA_WearAreavsPeriod<-permutest(mod2, pairwise = TRUE, permutations = 9999)

#To perform a site by site pairwise comparison of wear facet area composition.

pairmanWearareasite<-pairwise.perm.manova(Yperiod2,fact=WAF$Site,test=c("Pillai"),nperm=9999,p.method="fdr")

**Results**

The following tables (S1-S4) detail the results of the statistical analysis of the relationship between sex and wear facet expression in the material examined.

*S1 Table: Results of Type I PERMANOVA used to assess the relationship between sex and wear facet area composition among the Industrial assemblages examined.* ***Null hypothesis: relative wear facet area proportions did not differ significantly between the sexes during the Industrial period.***

|  | **Sum of Squares** | **Mean of Squares** | **DF** | **F** |  | **Probability (>F)** | **H_0_** |
| --- | --- | --- | --- | --- | --- | --- | --- |
| **Sex** | 0.00 | 0.00 | 1 | 0.00 | 0.00 | 1.00 | Not Rejected |
| **Residuals** | 47.59 | 0.54 | 88 |  | 1.00 |  |  |
| **Total** | 47.58 |  | 89 |  |  |  |  |

*S2 Table.* *Results of Independent sample t-tests to assess whether dip angles differed significantly between the sexes in the Industrial material examined. All data were normally distributed (Shapiro-Wilk p>0.05) and exhibited homogeneity of variance (Levene’s test p>0.05).* ***Null hypothesis: there were no significant differences in dip angle between the sexes for any wear facet type.*** **Bonferroni adjusted p-value=0.017.*

| **Wear Facet Type** | **BPI** | **LPI** | **PII** |
| --- | --- | --- | --- |
| **Female Mean (⁰)** | 31.39 | 32.3 | 27.24 |
| **Standard Deviation** | 9.43 | 7.58 | 8.46 |
| **Male Mean (⁰)** | 29.64 | 29.74 | 25.22 |
| **Standard Deviation** | 9.55 | 7.21 | 8.18 |
| **t-value** | 0.87 | 1.63 | 1.14 |
| **Degrees of Freedom** | 77.26 | 74.03 | 78.8 |
| **p value*** | 0.39 | 0.11 | 0.26 |
| **Effect size** | 0.19 | 0.35 | 0.24 |
| **95% CI Effect Size** | -0.23 to 0.61 | -0.08 to 0.79 | -0.18 to 0.67 |
| **Statistical Power** | 0.14 | 0.37 | 0.20 |
| **H_0_** | Not Rejected | Not Rejected | Not Rejected |

*S3 Table Results of Type I PERMANOVA assessing the relationship between sex and wear facet area proportions in the Medieval and early Post-Medieval periods.* ***Null Hypothesis: relative wear facet areas did not differ significantly between males and females in the Medieval and early Post-Medieval groups examined.***

|  | **Sum of Squares** | **Mean of Squares** | **Degrees of Freedom** | **F** | **p-value (>F)** | **H_0_** |
| --- | --- | --- | --- | --- | --- | --- |
| **Sex** | 0.46 | 0.46 | 1.00 | 1.27 | 0.28 | Not Rejected |
| **Residuals** | 26.80 | 0.36 | 74.00 |  |  |  |

*S4 Table Wilcoxon Rank Sum test results for the influence of sex of wear facet dip angle in the Medieval and early Post-Medieval periods. Non-parametric testing was used as the assumption of normality was violated (Shapiro-Wilk p>0.05).* ***Null hypothesis: dip angle values for the given wear facet type did not differ significantly between males and females in the pre-Industrial groups examined.***

| **Wear Facet Function** | **W** | **p-value** | **H_0_** |
| --- | --- | --- | --- |
| **BPI** | 943 | 0.85 | Not Rejected |
| **LPI** | 911.5 | 0.95 | Not Rejected |
| **PII** | 756 | 0.16 | Not Rejected |

**S1 Text References**

1. Kullmer O, Benazzi S, Fiorenza L, Schulz D, Bacso S, Winzen O. Technical note: Occlusal fingerprint analysis: Quantification of tooth wear pattern. Am J Phys Anthropol. 2009; 139(4): 600-605.
2. Fiorenza L, Benazzi S, Tausch J, Kullmer O, Bromage T, Schrenk F et al. Molar Macrowear Reveals Neanderthal Eco-Geographic Dietary Variation (Neanderthal Tooth Wear). PLoS ONE. 2011; 6(3): E14769.
3. Kullmer O, Menz U, Fiorenza L. Occlusal Fingerprint Analysis (OFA) reveals dental occlusal behavior in primate molars. In: Martin T, Koenigswald W.v, editors. Mammalian Teeth-Form and Function. Munich: Pfeil, 2020 pp.25-43.
4. Ulhaas L, Kullmer O, Schrenk F, Henke W. A new 3-d approach to determine functional morphology of cercopithecoid molars. Ann Anat. 2004; 186(5): 487-493.
5. Fiorenza L, Benazzi S, Oxilia G, Kullmer O. Functional relationship between dental macrowear and diet in Late Pleistocene and recent modern human populations. Int J Osteoarchaeol. 2018; 28(2): 153-161.
6. Janis CM. The correlation between diet and dental wear in herbivorous mammals, and its relationship to the determination of diets of extinct species. In: Boucot AJ, editors. Evolutionary Paleobiology of Behavior and Coevolution. Amsterdam: Elsevier Science; 1990. pp.241-259.
7. M‘Kirera F, Ungar PS. Occlusal Relief changes with molar wear in Pan troglodytes troglodytes and Gorilla gorilla gorilla. Am J Phys Anthropol. (2003); 60(2): 31-41.
8. Egozcue J, Pawlowsky-Glahn V, Mateu-Figueras G, Barceló-Vidal C. Isometric Logratio Transformations for Compositional Data Analysis. Math Geol. 2003; 35(3): 279-300.
