# Supplementary material for: A dental revolution: The association between occlusion and chewing behaviour
Source: PLoS One. 2021 Dec 15;16(12):e0261404. doi: 10.1371/journal.pone.0261404 (PMC8673603; doi:10.1371/journal.pone.0261404)
Supplement: S1 File — (PDF) [file pone.0261404.s004.pdf]

Open Government Licence
for public sector information

delivered by THE NATIONAL ARCHIVES

Back to The National Archives

You are encouraged to use and re-use the Information that is available under this licence freely and flexibly, with only a few conditions.

Using Information under this licence

Use of copyright and database right material expressly made available under this licence (the 'Information') indicates your acceptance of the terms and conditions below.

The Licensor grants you a worldwide, royalty-free, perpetual, non-exclusive licence to use the Information subject to the conditions below.

This licence does not affect your freedom under fair dealing or fair use or any other copyright or database right exceptions and limitations.

You are free to:

copy, publish, distribute and transmit the Information;

adapt the Information;

exploit the Information commercially and non-commercially for example, by combining it with other Information, or by including it in your own product or application.

You must (where you do any of the above):

acknowledge the source of the Information in your product or application by including or linking to any attribution statement specified by the Information Provider(s) and, where possible, provide a link to this licence;

If the Information Provider does not provide a specific attribution statement, you must use the following:

Contains public sector information licensed under the Open Government Licence v3.0.

If you are using Information from several Information Providers and listing multiple attributions is not practical in your product or application, you may include a URI or hyperlink to a resource that contains the required attribution statements.

These are important conditions of this licence and if you fail to comply with them the rights granted to you under this licence, or any similar licence granted by the Licensor, will end automatically.

Exemptions

This licence does not cover:

- personal data in the Information;
- Information that has not been accessed by way of publication or disclosure under information access legislation (including the Freedom of Information Acts for the UK and Scotland) by or with the consent of the Information Provider;
- departmental or public sector organisation logos, crests and the Royal Arms except where they form an integral part of a document or dataset;
- military insignia;
- third party rights the Information Provider is not authorised to license;
- other intellectual property rights, including patents, trade marks, and design rights; and
- identity documents such as the British Passport

Non-endorsement

This licence does not grant you any right to use the Information in a way that suggests any official status or that the Information Provider and/or Licensor endorse you or your use of the Information.

No warranty

The Information is licensed 'as is' and the Information Provider and/or Licensor excludes all representations, warranties, obligations and liabilities in relation to the Information to the maximum extent permitted by law.

The Information Provider and/or Licensor are not liable for any errors or omissions in the Information and shall not be liable for any loss, injury or damage of any kind caused by its use. The Information Provider does not guarantee the continued supply of the Information.

Governing Law

This licence is governed by the laws of the jurisdiction in which the Information Provider has its principal place of business, unless otherwise specified by the Information Provider.

Definitions

In this licence, the terms below have the following meanings:

'Information' means information protected by copyright or by database right (for example, literary and artistic works, content, data and source code) offered for use under the terms of this licence.

'Information Provider' means the person or organisation providing the Information under this licence.

'Licensor' means any Information Provider which has the authority to offer Information under the terms of this licence or the Keeper of Public Records, who has the authority to offer Information subject to Crown copyright and Crown database rights and Information subject to copyright and database right that has been assigned to or acquired by the Crown, under the terms of this licence.

'Use' means doing any act which is restricted by copyright or database right, whether in the original medium or in any other medium, and includes without limitation distributing, copying, adapting, modifying as may be technically necessary to use it in a different mode or format.

'You', 'you' and 'your' means the natural or legal person, or body of persons corporate or incorporate, acquiring rights in the Information (whether the Information is obtained directly from the Licensor or otherwise) under this licence.

About the Open Government Licence

The National Archives has developed this licence as a tool to enable Information Providers in the public sector to license the use and re-use of their Information under a common open licence. The National Archives invites public sector bodies owning their own copyright and database rights to permit the use of their Information under this licence.

The Keeper of the Public Records has authority to license Information subject to copyright and database right owned by the Crown. The extent of the offer to license this Information under the terms of this licence is set out in the UK Government Licensing Framework.

This is version 3.0 of the Open Government Licence. The National Archives may, from time to time, issue new versions of the Open Government Licence. If you are already using Information under a previous version of the Open Government Licence, the terms of that licence will continue to apply.

These terms are compatible with the Creative Commons Attribution License 4.0 and the Open Data Commons Attribution License, both of which license copyright and database rights. This means that when the Information is adapted and licensed under either of those licences, you automatically satisfy the conditions of the OGL when you comply with the other licence. The OGLv3.0 is Open Definition compliant.

Further context, best practice and guidance can be found in the UK Government Licensing Framework section on The National Archives website.

Go to the version 1 of the licence.
Go to the version 2 of the licence.
Go to the Welsh version of the licence.
